# PRDM12 in Health and Diseases

**DOI:** 10.3390/ijms222112030

**Published:** 2021-11-06

**Authors:** Monica Rienzo, Erika Di Zazzo, Amelia Casamassimi, Patrizia Gazzerro, Giovanni Perini, Maurizio Bifulco, Ciro Abbondanza

**Affiliations:** 1 Department of Environmental, Biological, and Pharmaceutical Sciences and Technologies, University of Campania “Luigi Vanvitelli”, 81100 Caserta, Italy; monica.rienzo@unicampania.it; 2 Department of Medicine and Health Sciences “V. Tiberio”, University of Molise, 86100 Campobasso, Italy; erika.dizazzo@unimol.it; 3 Department of Precision Medicine, University of Campania “Luigi Vanvitelli”, Via L. De Crecchio, 80138 Naples, Italy; ciro.abbondanza@unicampania.it; 4 Department of Pharmacy, University of Salerno, 84084 Fisciano, Salerno, Italy; pgazzerro@unisa.it; 5 Department of Pharmacy and Biotechnology, University of Bologna, 40126 Bologna, Italy; giovanni.perini@unibo.it; 6 Department of Molecular Medicine and Medical Biotechnologies, University of Naples “Federico II”, 80131 Naples, Italy; maubiful@unina.it

**Keywords:** cancer, cell metabolism, neurogenesis, pain perception, PRD-BF1 and RIZ homology domain containing gene family, *PRDM12*

## Abstract

PRDM12 is a member of the PRDI-BF1 (positive regulatory domain I-binding factor 1) homologous domain (PRDM)-containing protein family, a subfamily of Kruppel-like zinc finger proteins, controlling key processes in the development of cancer. *PRDM12* is expressed in a spatio-temporal manner in neuronal systems where it exerts multiple functions. PRDM12 is essential for the neurogenesis initiation and activation of a cascade of downstream pro-neuronal transcription factors in the nociceptive lineage. *PRDM12* inactivation, indeed, results in a complete absence of the nociceptive lineage, which is essential for pain perception. Additionally, PRDM12 contributes to the early establishment of anorexigenic neuron identity and the maintenance of high expression levels of pro-opiomelanocortin, which impacts on the program bodyweight homeostasis. PRDMs are commonly involved in cancer, where they act as oncogenes/tumor suppressors in a “Yin and Yang” manner. *PRDM12* is not usually expressed in adult normal tissues but its expression is re-activated in several cancer types. However, little information is currently available on *PRDM12* expression in cancers and its mechanism of action has not been thoroughly described. In this review, we summarize the recent findings regarding PRDM12 by focusing on four main biological processes: neurogenesis, pain perception, oncogenesis and cell metabolism. Moreover, we wish to highlight the importance of future studies focusing on the PRDM12 signaling pathway(s) and its role in cancer onset and progression.

## 1. Introduction

The human PRDM [PRDI-BF1 (Positive Regulatory Domain I-binding factor 1)] gene family consists of 19 members that encode for Kruppel-like zinc finger proteins, which share a conserved N-terminal PR domain, followed by several zinc finger domains that mediate sequence-specific DNA binding, protein–protein interactions and nuclear imports [1,2]. The PR domain is endowed with lysine methyltransferases (KMTs) activity; however, enzymatic activity is established only for a few family members [1,3,4,5]. Nonetheless, most of PRDM proteins (PRDMs) can directly or indirectly recruit histone-modifying enzymes [1]. As an example, PRDM1, PRDM5, PRDM6 and PRDM12 function with G9a histone methyltransferase (HMT) [6]. Although PRDMs could function differently in many contexts, several data propose that they act by regulating gene expression. Thus, PRDMs exert this role by either recognizing specific consensus sequences in promoters or acting as non-DNA binding cofactors [7,8]. Of note, PRDMs show a strong cell context dependency through the selection of different target gene promoters, binding sites and partners [1]. PRDMs are involved in the transduction of many cell signals and participate in many developmental processes, including the proliferation/differentiation switch, inflammation and metabolism control. For example, PRDM14 and PRDM1/BLIMP1 (B-lymphocyte-induced maturation protein 1), are essential for pluripotency maintenance in embryonic stem cells [9,10]; moreover, PRDM1 is identified as a master regulator of terminal B cell differentiation, whereas PRDM3 and PRDM16 play a key role in hematopoiesis and stem cell homeostasis [11]. In addition to that, PRDM16 contributes to lipid metabolism, adipocyte differentiation and cardiac development [12].

Recently, several reports indicated a pivotal role of multiple PRDMs in neuronal cell fate establishment, particularly in hindbrain and spinal cord formation. Prdm genes are dynamically expressed during the development of mouse and zebrafish nervous systems in a spatially and temporally restricted manner [11]. For instance, Prdm1 is relevant for neural crest and sensory neuron development in zebrafish embryos [13]. Prdm8 expression was highly regulated in a spatial-temporal manner during neuronal differentiation and/or specification; mechanistically, it forms a neuronal repressor complex with Bhlhb5, thus possibly directing neural development through the regulation of Cadherin-11 [14,15].

Prdm16 is also a crucial player in brain development and homeostasis; specifically, it controls neural stem cell maintenance and proliferation, intermediate progenitor proliferation, neuronal cell migration and ependymal cell differentiation, at least in part, by regulating genes involved in reactive oxygen species levels and the epigenetic states of its bound enhancers [16,17,18]. Similarly, PRDM12 is proposed as a “master regulator” of the nervous system regulatory cascade as it is required for midbrain cell differentiation [11,19,20,21]. PRDM12 could be detected in the mouse brain from embryonic day E10.5 and was expressed in different diencephalon and hypothalamus regions during embryogenesis, whereas, in humans, its expression was reported only in the peripheral nervous system [22].

PRDMs can also regulate the proliferation and differentiation of neuronal progenitors through epigenetic modifications. For instance, PRDM4 is a component of an epigenetic complex that can regulate the proliferative potential and modulate cell cycle progression in neural stem cells [23].

Some *PRDMs*, including *PRDM12*, were down-regulated in high infertility risk patients. The gonadotropin-releasing hormone agonist treatment used for the therapy of cryptorchidism, a risk factor for testicular cancer and infertility, induced these *PRDMs,* suggesting their involvement in these diseases [24].

The dysregulation of *PRDMs* is also involved in the onset and progression of several human cancers. Most *PRDM* genes express two main molecular variants, with one lacking the PR domain. These two isoforms, which can be generated by either alternative splicing or the alternative use of different promoters, play opposite roles in cancer [1,25,26]. Specifically, the full-length product (PR-plus) usually acts as a tumor suppressor, whereas the short isoform (PR-minus) functions as an oncogene; a clear example is provided by *PRDM2* [25,27]. Chromosomal rearrangements, mutations and/or the aberrant expressions of *PRDM2*, *PRDM3/MECOM* and *PRDM16* were reported in lymphoid/myeloid malignancies in accordance with their roles in the hematopoietic stem cell differentiation and homeostasis control [1,28]. *PRDM14* is overexpressed in approximately 25% of human lymphoid neoplasms [29]. Recently, in vivo studies showed that PRDM15 sustained cancer cell metabolism by regulating a transcriptional program that modulated the activity of the PI3K/AKT/mTOR pathway and glycolysis in B-cell lymphomas [30]. Currently, little information is available on *PRDM12* expression in cancers and its mechanism of action have not been described thus far.

Here, we summarize the current knowledge on *PRDM12* gene functions by dissecting their involvement in four main processes: neurogenesis, pain perception, cell metabolism and oncogenesis (see graphical abstract) [22,31]. Moreover, we attempt to provide insights for the future study of the signaling pathway(s) involving PRDM12 and to clarify its role in cancer onset and progression.

## 2. *PRDM12* Gene and Its Protein Product

**Figure 1:**
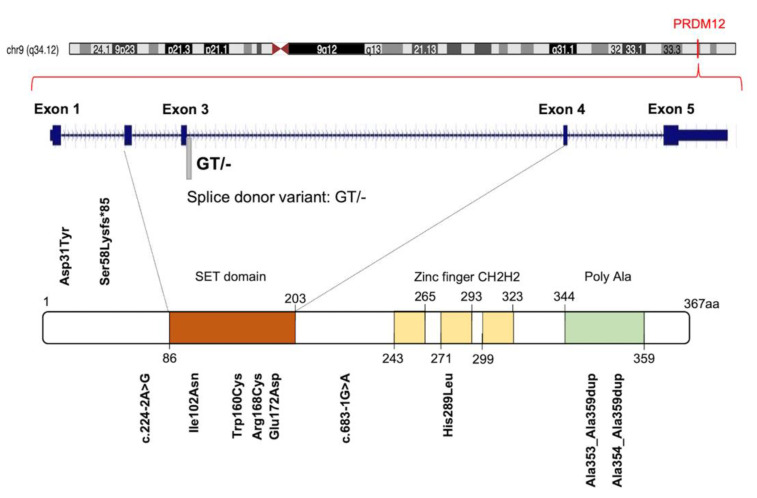
Graphic illustration of human *PRDM12* gene, protein and distribution of known congenital insensitivity to pain (CIP)-causing mutations. Figure shows a schematic representation of PRDM12 architecture: Su(var)3-9, Enhancer-of-zeste and Trithorax (SET) domain, three ‘classical’ zinc fingers (ZnF_C2H2), and a Poly Ala region. Amino acid numbering is reported. See text for details.

The human *PRDM12* gene is localized on chromosome 9 at 9q33-q34, according to the Entrez Gene [Gene ID: 59335]. It covers about 18.40 kb, from 133539981 to 133558370 (previous assembly, GRCh37.p13) or from 130664594 to 130682983 (current assembly, GRCh38.p13). The gene is also known as *HSAN8*, or *PFM9*, and it is structured in five coding exons and four introns on the sense strand (Figure 1). Unlike most of the *PRDM* family members, a unique *PRDM12* transcript of approximately 2478 bp is currently described [RefSeq accession: NM_021619.3], which encodes a single protein of 367 amino acids containing a PR domain (aa 86-203), three zinc finger domains C2H2-type (aa 243-265; 271-293; 299-323) and a C-terminal polyalanine tract (aa 344-359) [UniProt ID: Q9H4Q4] (Figure 1). The PRDM12 protein has a nuclear subcellular localization with a diffuse, lace-like pattern [32,33] and displays a restricted expression in adult human tissues (Figure S1).

**Figure 2:**
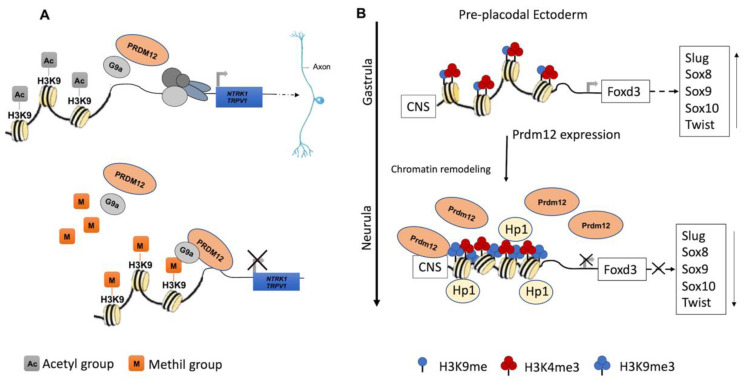
Recognized PRDM12 functions in neurogenesis. (**A**) PRDM12 lacks an intrinsic HKMTase activity, and it recruits, through its second zinc finger domain, the H3K9 methyltransferase G9a to dimethylate histone H3 at lysine 9 (H3K9me2), a repressive transcriptional mark. Particularly, PRDM12 regulates a nociceptor-specific transcriptional program, such as NTRK1/TRPV1. (**B**) In the pre-placodal ectoderm, *PRDM12* is expressed and specifically stimulates the trimethylation of histone H3 at lysine 9 (H3K9me3) on the *Foxd3* promoter to bind a conserved noncoding sequence (CNS). Additionally, Prdm12 inhibited the expression of different neural crest markers (Slug, Sox8, -9, -10 and Twist). A chromatin remodeling factor, HP1, recognizes the trimethylation of H3K9me3 and recruits other factors to convert euchromatin to heterochromatin.

PRDM12 lacks an intrinsic HKMTase activity, which is provided through the recruitment of the H3K9 methyltransferase, G9a, which dimethylates H3K9me2, a repressive transcriptional mark. The G9a recruitment mechanism is not completely clear. Indeed, it may occur through the PRDM12 zinc finger domain in mouse and Xenopus models, whereas it is not clear for zebrafish as the zinc finger domains are seemingly not required for this interaction (Figure 2A) [34,35,36]. In Xenopus, this activity is complemented by the histone H3 demethylase Kdm4a [31,34,37].

*PRDM12* gene is phylogenetically conserved during metazoan evolution, and it can also be found in some nonbilaterian phyla, such as sponges and cnidarians [38]. Additionally, *PRDM12* is one of the *PRDM* genes in which more ancient duplications occur; phylogenetic analyses strongly support the hypothesis of a duplication in the lineage leading to the Euteleostei ancestor, since several related species display more than one *PRDM12* gene, specifically the paralogs Prdm12a and Prdm12b [38]. Currently, additional 301 vertebrate sequences can be downloaded as orthologs of the human *PRDM12* gene from the NCBI web page [39].

## 3. Established *PRDM12* Functions: Neurogenesis

**Figure 3:**
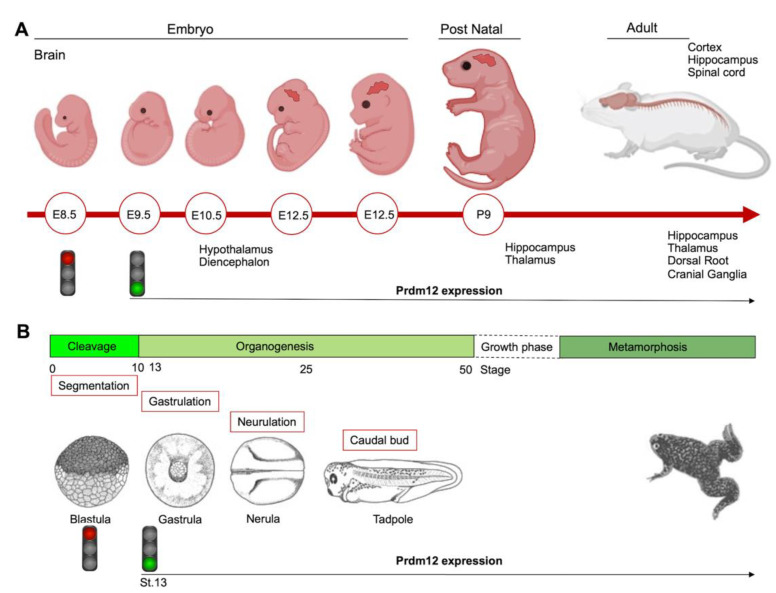
Schematics showing the Prdm12 expression data collected throughout mouse brain development and in different embryonal stages of *Xenopus*. (**A**) *Prdm12* was expressed from early neurogenesis (E9.5) in the developing spinal cord. Additionally, *Prdm12* can be detected weakly in the caudal forebrain and midbrain where it increases at E10.5 in precise neuronal progenitor areas. (**B**) *prdm12* expression was revealed during the early neurula stage of *Xenopus* embryos, specifically in the lateral pre-placodal ectoderm after the late gastrula stage (St.13).

To date, the role of PRDM12 in neurogenesis is well-established and corroborated by many studies both in in vitro cell cultures and in different animal models such as mouse, frog, chicken, zebrafish and drosophila, altogether confirming Prdm12 expression in the developing nervous systems. The first in vivo study reported that multiple genes in the *Prdm* family (*Prdm6*, -*8*, -*12*, -*13* and -*16*) were expressed in the developing mouse nervous systems in a spatially and temporally restricted manner [19]. Specifically, mouse *Prdm12* was expressed from early neurogenesis (E9.5) in the developing spinal cord and, weakly, in the caudal forebrain and midbrain, where it increased at E10.5 in precise neuronal progenitor areas where it could specify different neuronal subtypes (Figure 3A) [19]. With the development of telencephalon, Prdm12 was expressed in the ventricular zone in a lateral-to-medial graded manner. In the postnatal brain, it was expressed in the hippocampus, part of the hypothalamus, and in the thalamus, whereas, outside the brain, it was expressed in both the dorsal root and cranial ganglia. These findings implied its involvement in the patterning, differentiation and function of specific neurons, potentially regulated by the Notch-Hes pathway [19].

In P19 embryonal carcinoma cells, an in in vitro mouse model systems for neurogenesis, retinoic acid (RA) that prompted neural differentiation into neurons and glial cells, induced *Prdm12* expression, possibly through the regulation of a putative RA receptor (RAR)-beta response element. Additionally, *Prdm12* overexpression impaired P19 cell proliferation and increased the percentage of cells in the G1 phase accompanied by p27 upregulation. Furthermore, both the PR domain and zinc finger domains were required for the anti-proliferative activity of PRDM12. In contrast, *Prdm12* knockdown and *Prdm12* mutants resulted in an increased number of cells in a suspension culture of RA-induced neural differentiation [34]. Altogether, these results suggested that *Prdm12* was induced by the RA signaling and might control neural differentiation during development through p27 expression level regulation.

During the early neurula stage of *Xenopus* embryos, *prdm12* expression was also revealed in the lateral pre-placodal ectoderm after the late gastrula stage (st. 13), where it was regulated by both BMP and Wnt signaling (Figure 3B) [37]. Several gain- and loss-of-function experiments were approached to clarify the role of Prdm12 in early *Xenopus* development. *prdm12* overexpression through mRNA injection inhibited the expression of neural crest markers (Foxd3, Slug, Sox8, -9, -10 and Twist) via H3K9 trimethylation (H3K9me3) (Figure 2B). Otherwise, *prdm12* knockdown through an antisense morpholino oligomer (MO) inhibited the expression of presumptive trigeminal placode markers and expanded the neural crest region through a H3K9me3 level decrease in the *Foxd3* gene promoter (Figure 2B). Notably, the histone demethylase, Kdm4a, inhibited the expression of presumptive trigeminal placode markers producing a similar effect of *prdm12* knockdown. Accordingly, ChIP-qPCR analyses revealed that the expression of H3K9me3 on the Foxd3, Slug, and Sox8 promoters was inhibited by Kdm4a overexpression. Altogether, the mutual relationship between Prdm12 and Kdm4a indicated that the modification of the H3K9 methylation levels on the neural crest gene promoters by these two proteins would determine a demarcation line between the pre-placodal ectoderm and the neural crest region [37]. Interestingly, a recent analysis, performed to screen for zic1 targets in the midbrain region of *Xenopus*, revealed that *prdm12* was a downstream target of zic1 [21]. Zic1 is a highly conserved zinc finger transcription factor playing a critical role in the establishment of the nervous system; it is expressed on the lateral edge of the neural plate and in the dorsal neural tube [21]. Here, *prdm12* was expressed in the caudal forebrain, midbrain and hindbrain. Moreover, during embryonic development, *zic1* and *prdm12* were co-expressed in the same cell, with Zic1 controlling the expression of *prdm12* mediated by Wnt signaling during brain cell differentiation. Additionally, gain- and loss-of-function experiments revealed that *prdm12* was both necessary and sufficient to promote midbrain formation in the embryo [21].

In addition to the central nervous system, *prdm12* expression was also detected in the peripheral nervous system. The V1 interneurons are a class of inhibitory glycinergic neurons playing a conserved role in vertebrate locomotion; they originate from the spinal cord p1 domain and are characterized by the expression of Engrailed-1 (En1/Eng1). *prdm12b*, the zebrafish *prdm12* homolog, was expressed in the p1 domain of the neural tube at least partially in response to Sonic Hedgehog (Shh) signaling. Interestingly, *prdm12b* disruption led to the inappropriate dorsoventral patterning of the neural tube, depletion of the V1 interneurons and an impaired escape response in zebrafish. These data suggest that *prdm12b* is a key component of the genetic program required for motor circuit formation [40]. Likewise, in the frog embryos, *prdm12* was selectively expressed in p1 progenitors of the hindbrain and spinal cord; this restricted expression profile was also observed in the neural tube of chick embryos and in the ventral nerve cord of the larvae of the basal chordate amphioxus. Moreover, in frog, chicken and mice, *Prdm12* expression in the p1 domain progenitors of the caudal neural tube was dependent on RA signaling and Pax6 and it was repressed by *Dbx1* and *Nkx6-1/2* expressed in the adjacent p0 and p2 domains [35]. Functional studies in Xenopus and the genome-wide identification of molecular targets by RNA-seq and ChIP-Seq, revealed that the vertebrate Prdm12 acted as a general determinant of V1 cell fate, at least in part, by directly repressing *Dbx1* and *Nkx6* genes. Both the PR and zinc-finger domains of Prdm12 were required to exert this function; specifically, Prdm12 may act as a G9a-dependent repressor to induce En1. However, this activity was not found in the amphioxus, and differences in the C-terminal region of the protein, including the zinc-finger domains, may account for the differential functions of the amphioxus and vertebrate proteins. Overall, these findings indicated that Prdm12 could promote V1 interneurons through cross-repressive interactions with *Dbx1* and *Nkx6* genes. Interestingly, this function could be acquired after the split between the vertebrate and cephalochordate lineages [35]. Recently, the analysis of CRISPR/Cas9 *prdm12* mutants, recapitulating the phenotypes observed by MO-based approaches, has demonstrated that prdm12b acts as transcriptional repressor in zebrafish, and that it can interact with both EHMT2/G9a and Bhlhe22, a member of the basic Helix-Loop-Helix (bHLH) family, through its zinc-finger domain. However, *bhlhe22* function is not required for *eng1b* expression in vivo, suggesting that other bhlh genes could be involved during embryogenesis. This study also suggested that prdm12b is not only required to repress non-p1 fates, but also to promote p1 fates [36]. Additionally, a study in a mouse model revealed strong evidence that *Dbx1* and *Prdm12* expression was inhibited by both Pax3 and Pax7, two highly related transcription factors controlling the spatial organization of spinal differentiation [41]. Notably, another member of the Prdm family, PRDM13, was recently shown to be required for the restriction of *Prdm12* expression to the ventral neural tube during mouse embryogenesis [42]. In mouse *Prdm13* mutants, *Prdm12* was aberrantly expressed in the dorsal region, altering the identity of these neurons. Mechanistically, PRDM13 interacted with the genomic regions, overlapping those bound by neural bHLH factors and functions, by limiting the ability of these bHLH factors to activate enhancer-driven reporters. Specifically, PRDM13 repressed *Prdm12* in the dorsal neural tube via the inhibition of NEUROG1 and NEUROG2, which were likely to activate mouse *Prdm12* transcription through one enhancer localized more than 25 kb upstream of the ATG starting site [42].

PRDM12 could also function in the vagal sensory nervous system, to maintain visceral homeostasis. Indeed, transcriptome profiling performed to reveal differentially expressed genes between nodose and jugular C-fiber neurons detected *Prdm12* as preferentially expressed in mouse jugular vagal neurons [43].

Of note, PRDM12, together with ZIC1, ZIC2 and FABP7, were suggested as candidate targets for a vulnerability to cocaine addiction in mice. RNAseq and immunohistochemistry analysis revealed that these genes were downregulated in the nucleus accumbens (NAC), a key component of the reward circuitry, of Social Stress (S-S)-exposed juvenile mice, compared to control No Social Stress (NS-S) mice [44].

## 4. Established *PRDM12* Functions: Pain Perception

Several studies established that *PRDM12* was essential for human pain perception [31,45]. The sensation of pain is a conserved, protective mechanism essential for the preservation of the body’s functional integrity. Acute pain, caused by damage and mechanical, chemical, or thermal stimuli, is perceived by a specialized group of peripheral neurons, called nociceptors [31]. The key molecular regulators necessary for the development and initiation of pain-sensing neurons remain largely unknown. Nevertheless, many recent insights about the molecular basis of the pain sensitivity system were provided by studying painless genetic disorders and through the detection of their responsible genes, such as *NGF* encoding the nerve growth factor β, and *NTRK1* encoding its receptor, the tropomyosin receptor kinase A (TRKA). Several research groups, investigating the PRDM12 role in vertebrate nervous system patterning, discovered that *PRDM12* was mutated in families with pain perception alterations (Table S1, Figure 1) [31,46].

Initially, 10 different homozygous mutations in *PRDM12* were identified in subjects from 11 families with a congenital insensitivity to pain (CIP), a type of hereditary sensory and autonomic neuropathy (HSAN), which is a clinically and genetically heterogeneous group of inherited neuropathies predominantly affecting peripheral sensory and autonomic neurons [32]. Most of the variants were missense mutations, despite the revelation of a splice-site mutation, a frame-shift mutation and an 18-alanine-repeat mutation (general population contains a maximum of 14-alanine in this polymorphic site; Figure 1 and Table S1). Heterozygote carriers were asymptomatic with a normal pain perception. To determine whether these mutations could cause developmental defects in the sensory neurons, committed to becoming nociceptors, the expression of *PRDM12* during embryogenesis and the differentiation in various in vivo models (mouse, Xenopus and human iPSC derived sensory neurons) was explored. Prdm12 was expressed in nociceptors and their progenitors and participated in the development of sensory neurons [32]. Moreover, the CIP-associated *PRDM12* mutations impaired the histone-methylation capacity [32]. In an independent study, Prdm12 was also investigated as a key regulator of sensory neuronal specification in *Xenopus* [47]. In this case, the modeling analysis of human *PRDM12* mutations causing HSAN revealed a remarkable conservation of the mutated residues during evolution. As shown by RNAseq analyses, the expression of wild-type human *PRDM12* in *Xenopus* induced the expression of several sensory neuronal markers including Islet1 and Tlx3; in contrast, embryos treated with *PRDM12* MO or *PRDM12* mutants displayed reduced levels of these markers [47]. In *Drosophila*, the Hamlet gene was identified as the functional *PRDM12* homolog that controls nociceptive behavior in sensory neurons. Interestingly, the ectopic expression of human *PRDM12* mutants in *Drosophila* nociceptor neurons impaired pain perception, thus supporting the idea that *PRDM12* was an evolutionary, conserved, master regulator of sensory neuronal specification that played a critical role in pain perception [47]. In addition to that, RNAseq analyses of human patient fibroblasts with *PRDM12* mutations disclosed the possible downstream target genes. Among them, the gene-encoding, thyrotropin-releasing, hormone-degrading enzyme (*TRHDE*) was revealed; its substrate, TRH, was previously found to affect pain in human and rodents. *TRHDE* knockdown in Drosophila sensory neurons resulted in an altered cellular morphology and impaired nociception. These findings also added to our knowledge that novel molecules and pathways controlled evolutionary, conserved nociception [47].

Other papers reported *PRDM12* mutations in CIP and similar pathological conditions [32]. Some authors suggested that *PRDM12*-CIP was a phenotypically distinct form of ‘Congenital Insensitivity to Pain’ [48]. A case report highlighted a novel *PRDM12* mutation presenting as early-onset, autosomal-recessive, sensory polyneuropathy; congenital insensitivity to pain; touch and temperature; global developmental delay and early loss of muscle stretch reflexes [49]. A further study presented the manifestations and dental management of a patient with HSAN-VIII, harboring the homozygous mutation c.516G>C (p. Glu172Asp) in the *PRDM12* gene (Figure 1) [50].

Additionally, the term ‘midface toddler excoriation syndrome’ (MiTES) was proposed for a distinctive, localized skin condition sometimes occurring in the context of a mild neurological deficit or congenital insensitivity to pain [51]. MiTES may reflect a limited or early manifestation of CIP [52]. Four out of five children, from four families, with facial lesions typical of MiTES, showed homozygous or heterozygous pathogenic expansions of the PRDM12 polyalanine tract. This finding extended the phenotypic spectrum of *PRDM12* mutations, which usually caused HSAN-VIII, characterized by mutilating, self-inflicted wounds of the extremities, lips and tongue. By contrast, MiTES showed severe midfacial lesions with little, if any, evidence of generalized pain insensitivity. Again, this condition is most likely genetically heterogeneous [52].

To date, the number of reported cases of both syndromes, HSAN-VIII and MiTES harboring *PRDM12* mutations has expanded, and it is likely to increase further in the future due to the advancement of sequencing strategies [53,54,55,56,57,58,59,60].

Overall, these genetic studies strongly support the model that PRDM12 could participate in sensory neuron development. However, only recently, the combined loss- and gain-of-function approaches in mouse and chicken have clearly demonstrated that PRDM12 is essential for determining the nociceptive lineage from neural crest cell progenitors [61]. In the absence of *PRDM12*, specific neuronal progenitors completely failed to maintain the expression of neurogenin (*Ngn1*), a fundamental factor for the generation of the nociceptive lineage, and to activate the downstream pro-neuronal genes *NEUROD1*, *BRN3A*, and *ISL1*. The loss of neurogenin expression was concomitant with a decrease in the number of progenitors and their proliferation. Similarly, *PRDM12* loss also failed to repress alternative fates in progenitor cells implying that the mechanism of nociceptive fate commitment is molecularly defined in precursors [61]. Constitutive and conditional *Prdm12* knock-out, mouse models and gain-of-function approaches in *Xenopus* and human iPSCs, showed that PRDM12 could regulate a nociceptor-specific, transcriptional program in sensory ganglia [62]. Specifically, PRDM12 cooperates with the proneural factors NGN1/2 to promote the activation and maintenance of TRKA (*Ntrk1*) expression and other nociceptive markers such as *TrpV1*, allowing nociceptor survival and differentiation during development (Figure 2A). Aside from *Ntrk1* expression at E11.5, PRDM12 contributed to the transcription regulation of several other nociceptive target genes such as *Nhlh1*, *Brn3a*, and *Neurod1* [62].

A very recent study investigated how *Prdm12* deletion during development or adulthood could affect nociception by employing tissue- and temporal-specific knockout mouse models. Results showed that constitutive *Prdm12* loss caused deficiencies in proliferation during sensory neurogenesis. More interestingly, the conditional knockout from dorsal root ganglia during embryogenesis caused defects in nociception, whereas in adult dorsal root ganglia, PRDM12 was unnecessary for most pain-sensation and injury-induced hypersensitivity. Transcriptome analysis also emphasized different expression patterns between adult and embryonic *Prdm12* knockout and that PRDM12 could be a transcriptional activator in the adult. Notably, these findings strongly suggest that PRDM12 may have different functions during the developmental stages [63]. Likewise, several developmental and adult-onset deletion mouse models confirmed the PRDM12 requirement for the genesis and function of nociceptors, whereas its proper expression was no longer essential for survival or needed for the established CNS functions, but it was still required for nociceptive responses [59]. Moreover, developmental or adult-onset deletion of *Prdm12* caused different effects on downstream gene expressions, supporting the hypothesis that PRDM12 regulates distinct, age-dependent transcriptional patterns [59].

The upstream genes and signals controlling *PRDM12* expression in developing sensory ganglia still remain to be addressed. It could be speculated that PRDM12, essential for TrkA initiation, is also a target of NGF-TrkA signaling, considering that, in adult mice and humans, NGF signaling induces nociceptor sensitization leading to chronic pain states [64] and *PRDM12* is highly expressed in mature nociceptors. Interestingly, *PRDM12* expression increased significantly (by 1000 times) when skin-derived precursor cells (a subtype of neural crest stem cells that persist in certain adult tissues such as the skin) were induced in vitro to differentiate into sensory neurons by several molecules, including NGF, which prompted the upregulation of neurogenins [65].

Moreover, both other partners of PRDM12 constituting the transcriptional complex, which epigenetically regulated gene expression in developing nociceptors, as well as the PRDM12 transcriptional targets need to be identified. Overall, these studies suggest that pharmacotherapies targeting this pathway, or the epigenetic mechanisms controlled by PRDM12, and could be a promising strategy in the treatment of chronic pain conditions.

The supplementary understandings of the identity of the potential PRDM12 interactors and the transcriptional, and epigenomic changes in the sensory neuron progenitors upon *PRDM12* manipulation, will be relevant in understanding the *PRDM12* gene regulation during the generation of the nociceptive lineage. The further comprehension of the involved molecular mechanisms will provide key insights into how sensory neuron diversity is generated and may provide genetic tools to induce a desired neuronal lineage in stem cell engineering.

## 5. Exploring Novel *PRDM12* Functions: Cancer

Although little is known about the function of PRDM12 in oncogenesis, previous studies showed that *PRDM12* might act as a tumor suppressor gene in human chronic myeloid leukemia (CML) [66,67,68]. In approximately 15% of CML patients, deletions occur on the derivative chromosome 9 [der(9)] within a region containing the PRDM12 gene. The *PRMD12* disruption could prompt the aggressive phenotype and the observed short survival [67]. However, further investigation is warranted to elucidate its role in the CML pathogenesis.

Our pan-cancer meta-analysis based on The Cancer Genome Atlas (TCGA) data showed that *PRDM12* was upregulated in several cancer types: colon, breast, kidney, colon, lung, liver, thyroid, ovary and prostate cancers, suggesting that it could represent a putative tumor marker [25]. These findings indicate that *PRDM12* is not expressed in adult normal tissues. Accordingly, *PRDM12* expression was described only in dorsal root ganglia but not in other adult tissues [32]. Additionally, the integrated analysis of abnormalities of HMTs encoding genes in prostate cancer from TCGA, identified a role for *PRDM12* in the pathogenesis of this cancer type [69]. *PRDM12* gene amplification induced an mRNA expression level increase in cancer cells compared to adjacent normal ones. Moreover, *PRDM12* gene expression showed a significantly positive correlation with the Gleason’s score. These findings indicated that *PRDM12* expression level alterations in prostate cancer tissue samples could have a prognostic value [69]. Similarly, in a recent study, somatic copy number alterations were also found for *PRDM12* in stomach adenocarcinoma samples [70]. However, currently, no studies have identified the mechanism by which PRDM12 could participate in oncogenesis. Moreover, as mentioned above, unlike the other PRDM family members, a unique transcript is known for *PRDM12* gene signifying, which means that the well-known “Yin and Yang” mechanism cannot function [28]. Indeed, it is recognized that different isoforms exist for almost all PRDM family members, and they play opposite roles in cancer; this duality is termed the ‘Yin and Yang’ mechanism, typical of PRDMs and involving a complex regulation of alternative splicing or alternative promoter usage, to generate full-length or PR-lacking isoforms [1,26,27,28].

The mutational profiling analyses of *PRDM12* gene across human cancers revealed 72 mutations, 30 of which were detrimental somatic mutations (frameshift, in-frame deletions, stop gained and start lost mutations; splice site, UTR, and intron variants). Interestingly, those mutations were significantly enriched in the PR domain of PRDM12 [25]. In particular, PRDM12 was frequently mutated in a splice donor site in a region coding for the PR domain in different tumor types (breast cancer; colon adenocarcinoma; kidney renal clear cell carcinoma; lung adenocarcinoma; pancreatic adenocarcinoma; prostate adenocarcinoma; skin cutaneous melanoma; thyroid carcinoma; uterine corpus endometrial carcinoma) indicating this position as a possible mutational hotspot site (Figure 1) [25].

Although preliminary lines of evidence suggest that PRDM12 is endowed with a tumor-promoting function, several aspects should be investigated to define the role of PRDM12 in cancer. Cancer tissue specimen analysis by immunohistochemistry could be useful to establish if a correlation among PRDM12 expression, with grading, tumor size, biomarkers serum levels, tumor vascular invasion, overall survival and prognosis, exists. Additionally, in vitro studies should be carried out to investigate the functional role of PRDM12. Overexpression and silencing experiments should be performed to assess its role in cell viability, cell death and proliferation, and cell migration and invasion. Finally, a transcriptome profiling analysis of both PRDM12 overexpressing and silenced cells could reveal PRDM12 target genes and the involved regulated pathways, thus clarifying its mechanism of action. The PRDM12 function elucidation could provide new insights useful for the discovery of novel therapeutic approaches.

## 6. Exploring Novel *PRDM12* Functions: Cell Metabolism

**Figure 4:**
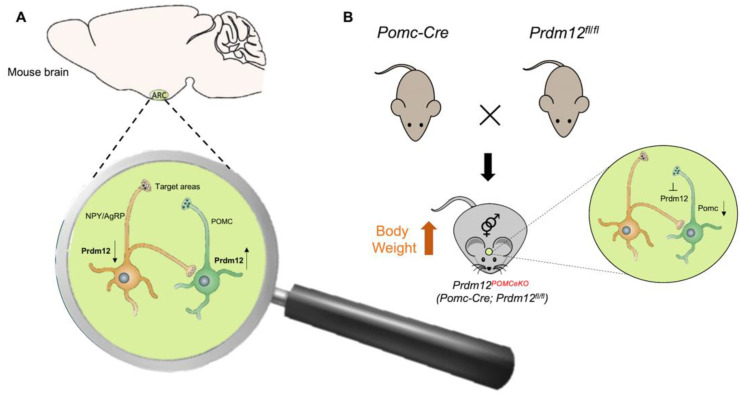
Possible mechanism of PRDM12 in POMC neurons. (**A**) PRDM12 expression level in POMC and NPY/AgRP neurons in the arcuate nucleus of the hypothalamus (ARH) in mouse brain. (**B**) To study the role of PRDM12 specifically in postmitotic POMC neurons, *Pomc-Cre* mice were bred with *Prdm12fl/fl* mice to generate *Pomc-Cre*; in *Prdm12fl/fl* mice *Prdm12* was selectively deleted in embryonic POMC neurons (designated as Prdm12POMCeKO mice). The expression of Npy and Agrp remained unaffected. Prdm12POMCeKO mice lacking Prdm12 selectively from POMC neurons showed a considerable reduction in *Pomc* mRNA levels that led to severe obesity.

An interesting and unexpected PRDM12 role in metabolism was recently demonstrated [71]. Food intake and energy balance regulation depends on the arcuate nucleus of the hypothalamus (ARH), consisting of two distinct neuronal populations: the pro-opiomelanocortin (POMC)-expressing neurons and the neuropeptide Y/agouti-related peptide (NPY/AgRP)-expressing neurons. POMC and NPY/AgRP expressing neurons derive from the same hypothalamic progenitor but have opposing effects on food intake, being the first anorexigenic (POMC) and the last orexigenic (NPY/AgRP). The cell-type-specific transcriptome profiles of developing POMC and NPY/AgRP neurons in mice revealed that POMC and NPY/AgRP cell fates are specified and maintained by distinct intrinsic factors. The transcription regulator PRDM12 was selectively enriched in POMC neurons but absent in NPY/AgRP neurons (Figure 4A) [71]. PRDM12 plays an essential role in the early establishment of hypothalamic melanocortin neuron identity and function. PRDM12, indeed, is co-expressed with POMC in mouse neurons of the ARH from the onset of *Pomc* expression at E10.5 and throughout the lifespan [22]. The selective ablation of *Prdm12* from ISL1 neurons greatly reduced *Pomc* expression in the developing hypothalamus, demonstrating that it was essential for the onset and later maintenance of *Pomc* expression. PRDM12 integrates a distinctive set of transcriptional regulators, including NKX2.1 and ISL1, to dictate the neuronal-specific expression of ARH *Pomc*. Moreover, PRDM12 acts to program bodyweight homeostasis, maintaining the hypothalamic *Pomc* mRNA expression level. Adult mice of both sexes selectively lacking *Prdm12* from POMC neurons, showed a considerable reduction in *Pomc* mRNA levels that led to an amplified food intake, adiposity and bodyweight gain, as well as early-onset obesity that recapitulated symptoms of human POMC deficiency (Figure 4B) [22,71].

Additional studies should be performed to define the PRDM12 role in human metabolic diseases. The study population could be useful to define whether *PRDM12* polymorphisms represent a risk factor for obesity under permissive environmental conditions. It is conceivable that *PRDM12* polymorphisms generating hypomorphic alleles could impair ARH *Pomc* expression, and thus compromise food intake and energy balance control. Moreover, the disclosure of the PRDM12 mechanism of action in food intake and energy balance could have a relevant impact for the identification of new strategies to counteract obesity.

## 7. Conclusions

Neuronal cell fate specification is orchestrated by several fine-tuned molecular mechanisms in which several transcription factors are involved. Developmental studies uncovered the highly tissue-specific expression of *PRDM12* as well as its involvement in neuronal lineage specification. Indeed, emerging evidence suggests that PRDM12 cooperates with several proteins to regulate a critical set of genes required for the commitment of neuronal progenitors. Moreover, the mechanisms underlying G9a recruitment and function should be elucidated given the recent progress on the use of molecules targeting this histone methyltransferase for the therapy of many cancers and other human diseases [72]. Accordingly, *PRDM12* is not expressed in adult normal tissues, even though its expression is re-activated in several cancer types. However, the upstream regulatory signals from oncodrivers or signaling pathways establishing oncogenic transcriptional programs, or supporting chromatin remodeling towards a pro-oncogenic phenotype involving PRDM12, should be addressed. Targeting PRDM12 and its transcriptional program could have a high therapeutic potential, representing a promising strategy to overcome resistance and selectively target cancer cells. Thus, the further elucidation of involved molecular mechanisms will also provide new tools for therapy.

Overall, studies aimed to identify the potential PRDM12 interactors, their transcriptional and epigenetic activities, and downstream cellular functions are necessary to clarify how PRDM12 and its interactors function in health and diseases.

## Supplementary Materials

The following are available online at https://www.mdpi.com/article/10.3390/ijms222112030/s1.
