# Supplementary material for: PRDM12 in Health and Diseases
Source: Int J Mol Sci. 2021 Nov 6;22(21):12030. doi: 10.3390/ijms222112030 (PMC8585061; doi:10.3390/ijms222112030)
Supplement: Supplementary file 1 [file ijms-22-12030-s001.zip › Supplementary_files_IJMS2021/Table_S1.pdf]

Table S1. PRDM12 ClinVar genetic disease variations for Neuropathy, Hereditary Sensory and Autonomic, Type Viii

NM_021619.3 (**PRDM12**) variants	**Variant Type**	**Significance**	**ClinVar Id**	**dbSNP ID**	**GRCh37 Pos**	**GRCh38 Pos**	**Ref.**
c.305T>A (p.Ile102Asn)	SNV	Pathogenic	253119	rs879255636	9:133542076-133542076	9:130666689-130666689	26
c.91G>T (p.Asp31Tyr)	SNV	Pathogenic	253120	rs879255637	9:133540131-133540131	9:130664744-130664744	26, 39
c.516G>C (p.Glu172Asp)	SNV	Pathogenic	253121	rs755205487	9:133543646-133543646	9:130668259-130668259	26,39
c.866A>T (p.His289Leu)	SNV	Pathogenic	253122	rs879255638	9:133556818-133556818	9:130681431-130681431	26
c.1041_1043CGC[18] (p.Ala354_Ala359dup)	short repeat	Likely pathogenic	848182		9:133556992-133556993	9:130681605-130681606	26
c.620A>G (p.Asn207Ser)	SNV	Uncertain significance	849430		9:133553965-133553965	9:130678578-130678578	-
c.1028A>C (p.His343Pro)	SNV	Uncertain significance	854535		9:133556980-133556980	9:130681593-130681593	-
c.1041_1043CGC[17] (p.Ala355_Ala359dup)	short repeat	Uncertain significance	848087		9:133556992-133556993	9:130681605-130681606	-
c.440G>A (p.Arg147His)	SNV	Uncertain significance	568097	rs139807684	9:133543570-133543570	9:130668183-130668183	-
c.1039C>G (p.Leu347Val)	SNV	Uncertain significance	568099	rs1564249877	9:133556991-133556991	9:130681604-130681604	-
c.1093A>G (p.Met365Val)	SNV	Uncertain significance	572708	rs1344661944	9:133557045-133557045	9:130681658-130681658	-
c.607G>A (p.Gly203Arg)	SNV	Uncertain significance	582663	rs375887892	9:133553952-133553952	9:130678565-130678565	-
c.831C>G (p.Asn277Lys)	SNV	Uncertain significance	572393	rs995100590	9:133556783-133556783	9:130681396-130681396	-
c.995C>T (p.Ala332Val)	SNV	Uncertain significance	577727	rs773010364	9:133556947-133556947	9:130681560-130681560	-
c.1003C>A (p.Pro335Thr)	SNV	Uncertain significance	571567	rs1564249834	9:133556955-133556955	9:130681568-130681568	-
c.1022_1027ACGCGC[3] (p.341_342HA[3])	short repeat	Uncertain significance	571070	rs1288821918	9:133556970-133556971	9:130681583-130681584	-

c.979A>G (p.Ser327Gly)	SNV	Uncertain significance	660300		9:133556931-133556931	9:130681544-130681544	-
c.1041_1043CGC[11] (p.Ala359del)	short repeat	Uncertain significance	648392		9:133556993-133556995	9:130681606-130681608	-
c.499G>A (p.Ala167Thr)	SNV	Uncertain significance	475815	rs1554752141	9:133543629-133543629	9:130668242-130668242	-
c.1044_1045insACC (p.Ala349_Ala350insThr)	insertion	Uncertain significance	475804	rs1429038624	9:133556994-133556995	9:130681607-130681608	-
c.1041_1043CGC[5] (p.Ala353_Ala359del)	short repeat	Uncertain significance	475805	rs752427775	9:133556993-133557013	9:130681606-130681626	-
c.1041_1043CGC[15] (p.Ala357_Ala359dup)	short repeat	Uncertain significance	475810	rs752427775	9:133556992-133556993	9:130681605-130681606	-
c.1041_1043CGC[19] (p.Ala353_Ala359dup)	short repeat	Uncertain significance	475806	rs752427775	9:133556992-133556993	9:130681605-130681606	26
c.1034_1039dup (p.Pro345_Ala346dup)	duplication	Uncertain significance	542460	rs1298266062	9:133556980-133556981	9:130681593-130681594	-
c.1041_1043CGC[8] (p.Ala356_Ala359del)	short repeat	Likely benign	542462	rs752427775	9:133556993-133557004	9:130681606-130681617	-
c.995C>A (p.Ala332Glu)	SNV	Likely benign	475818	rs773010364	9:133556947-133556947	9:130681560-130681560	-
c.426G>A (p.Glu142=)	SNV	Likely benign	475814	rs139493961	9:133543556-133543556	9:130668169-130668169	-
c.711T>G (p.Ala237=)	SNV	Likely benign	475816	rs754277042	9:133556663-133556663	9:130681276-130681276	-
c.570+1GT[10]	short repeat	Likely benign	542461	rs138789124	9:133543700-133543701	9:130668313-130668314	-
c.540C>T (p.Ile180=)	SNV	Likely benign	702870		9:133543670-133543670	9:130668283-130668283	-
c.1041_1043CGC[13] (p.Ala359dup)	short repeat	Benign	475813	rs752427775	9:133556992-133556993	9:130681605-130681606	-
c.1041_1043CGC[9] (p.Ala357_Ala359del)	short repeat	Benign	475809	rs752427775	9:133556993-133557001	9:130681606-130681614	-
c.1041_1043CGC[6] (p.Ala354_Ala359del)	short repeat	Benign	475807	rs752427775	9:133556993-133557010	9:130681606-130681623	-

c.1041_1043CGC[7] (p.Ala355_Ala359del)	short repeat	Benign	475808	rs752427775	9:133556993-133557007	9:130681606-130681620	-
855G>A (p.Thr285=)	SNV	Benign	475817	rs76175818	9:133556807-133556807	9:130681420-130681420	-
c.1041_1043CGC[14] (p.Ala358_Ala359dup)	short repeat	Benign	475812	rs752427775	9:133556992-133556993	9:130681605-130681606	-
c.1041_1043CGC[16] (p.Ala356_Ala359dup)	short repeat	Benign	542463	rs752427775	9:133556992-133556993	9:130681605-130681606	-
c.1041_1043CGC[10] (p.Ala358_Ala359del)	short repeat	Benign	475811	rs752427775	9:133556993-133556998	9:130681606-130681611	-
**PRDM12**, (GCC)n REPEAT EXPANSION	NT expansion	Pathogenic	253118	rs752427775	9:133556993-133556995	9:130681606-130681608	26
NC_000009.12:g.130677193_130686925del	deletion	Pathogenic	689497		9: 133552559-133562291	9:130677172-130686904	-
NC_000009.12:g.(?_130678509)_(130678660_?)del	deletion	Uncertain significance	584194		9:133553896-133554047	9:130678509-130678660	-
NC_000009.12:g.(?_130664644)_(130681679_?)dup	duplication	Uncertain significance	833047		9:133540031-133557066		-

SNV= single nucleotide variant
