# Supplementary material for: PRDM12 in Health and Diseases
Source: Int J Mol Sci. 2021 Nov 6;22(21):12030. doi: 10.3390/ijms222112030 (PMC8585061; doi:10.3390/ijms222112030)
Supplement: Supplementary file 1 [file Figure S1.pdf › Supplementary_files_IJMS2021/Figure S1.pdf]

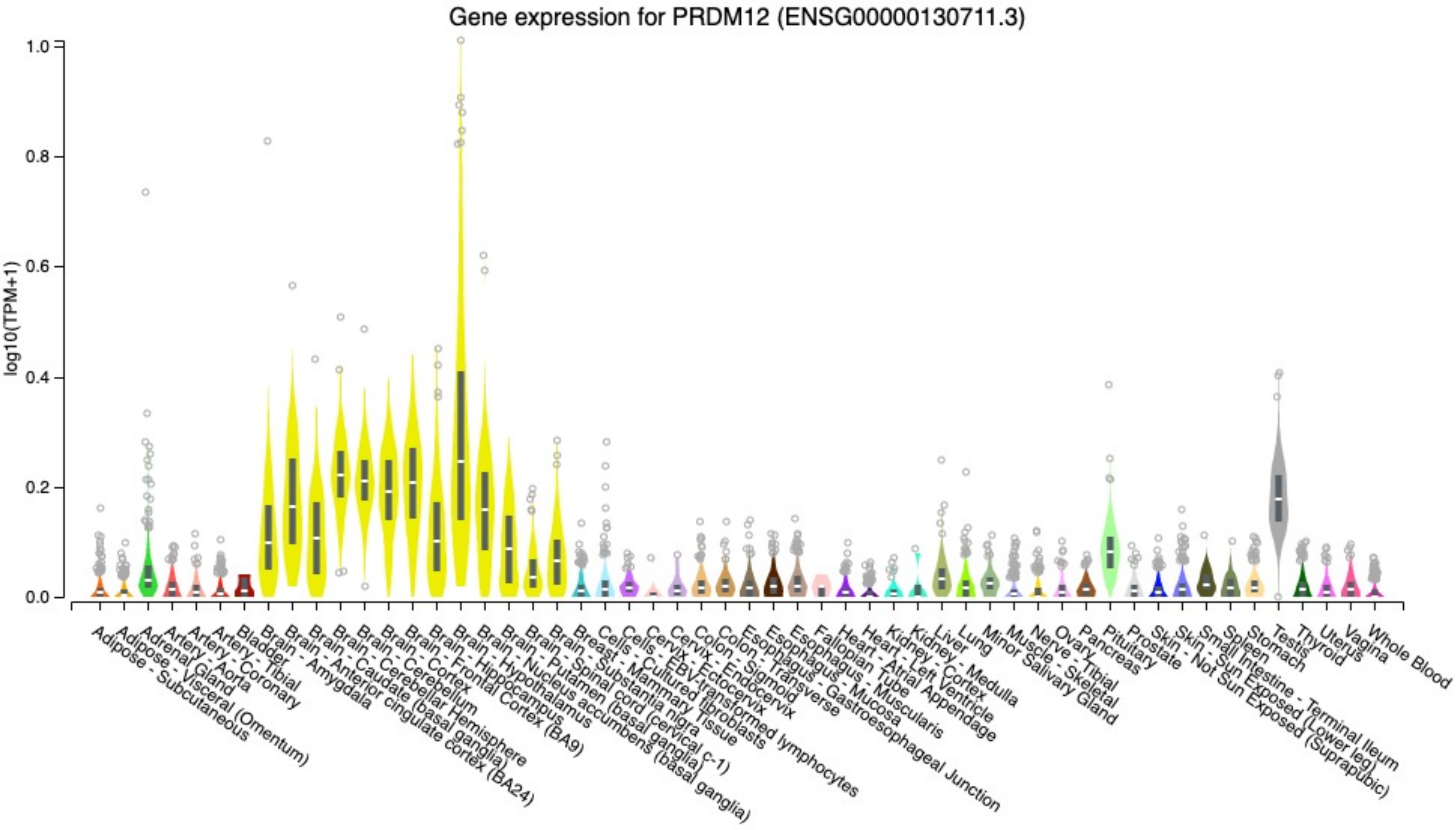

Figure S1: Expression profile of *PRDM12* gene from Genotype-Tissue Expression Portal (GTEx) in multiple normal tissue. Gene expression values in the different tissues (*x*-axis) are shown as transcript per million (Log10) on the *y*-axis.
